# Development of Highly Efficient, Glassy Carbon Foam Supported, Palladium Catalysts for Hydrogenation of Nitrobenzene

**DOI:** 10.3390/nano11051172

**Published:** 2021-04-29

**Authors:** Ádám Prekob, Mahitha Udayakumar, Gábor Karacs, Ferenc Kristály, Gábor Muránszky, Anett Katalin Leskó, Zoltán Németh, Béla Viskolcz, László Vanyorek

**Affiliations:** 1 Institute of Chemistry, University of Miskolc, H-3515 Miskolc-Egyetemváros, Hungary; kempadam@uni-miskolc.hu (Á.P.); kemudaya@uni-miskolc.hu (M.U.); kemmug@uni-miskolc.hu (G.M.); bela.viskolcz@uni-miskolc.hu (B.V.); kemvanyi@uni-miskolc.hu (L.V.); 2 Advanced Materials and Intelligent Technologies Higher Education and Industrial Cooperation Centre, University of Miskolc, H-3515 Miskolc-Egyetemváros, Hungary; 3 MTA-ME Materials Science Research Group, ELKH, H-3515 Miskolc-Egyetemváros, Hungary; femkg@uni-miskolc.hu; 4 Institute of Mineralogy and Geology, University of Miskolc, H-3515 Miskolc-Egyetemváros, Hungary; askkf@uni-miskolc.hu; 5 Institute of Energy and Quality Affairs, University of Miskolc, H-3515 Miskolc-Egyetemváros, Hungary; kkklesko@uni-miskolc.hu

**Keywords:** glassy carbon foam, nitrobenzene, palladium, conversion

## Abstract

Glassy carbon foam (GCF) catalyst supports were synthesized from waste polyurethane elastomers by impregnating them in sucrose solution followed by pyrolysis and activation (AC) using N_2_ and CO_2_ gas. The palladium nanoparticles were formed from Pd(NO_3_)_2_. The formed palladium nanoparticles are highly dispersive because the mean diameters are 8.0 ± 4.3 (Pd/GCF), 7.6 ± 4.2 (Pd/GCF-AC1) and 4.4 ± 1.6 nm (Pd/GCF-AC2). Oxidative post-treatment by CO_2_ of the supports resulted in the formation of hydroxyl groups on the GCF surfaces, leading to a decrease in zeta potential. The decreased zeta potential increased the wettability of the GCF supports. This, and the interactions between –OH groups and Pd ions, decreased the particle size of palladium. The catalysts were tested in the hydrogenation of nitrobenzene. The non-treated, glassy-carbon-supported catalyst (Pd/GCF) resulted in a 99.2% aniline yield at 293 K and 50 bar hydrogen pressure, but the reaction was slightly slower than other catalysts. The catalysts on the post-treated (activated) supports showed higher catalytic activity and the rate of hydrogenation was higher. The maximum attained aniline selectivities were 99.0% (Pd/GCF-AC1) at 293 K and 98.0% (Pd/GCF-AC2) at 323 K.

## 1. Introduction

The reduction of nitrobenzene is a commonly used process for aniline production [1,2,3,4,5]. About 70% of aniline is used for polyurethane production and the rest is applied in the pharmaceutical and dye industry. Catalytic hydrogenation is an established method for aniline production in the chemical industry. The hydrogenation of nitro compounds is a complicated reaction due to the formation of many intermediates and by-products; thus, the production of a catalytically active and selective catalyst is a challenge for catalyst researchers [6,7]. Carbon-based catalysts (mainly activated carbon or carbon nanotubes) are commonly used for this purpose, taking advantage of their high specific surface area, chemical and mechanical resistance, and good adsorption properties [8,9,10,11]. Both noble and transition metals are used as catalytically active metals, and their combinations and alloys are also applicable [12,13,14,15].

Carbon black (CB) is a popular catalyst support due to its properties and low price. It has already been shown that it is suitable for the preparation of a hydrogenation catalyst with high conversion and selectivity [16,17]. However, it is of interest to explore if a three-dimensional form of amorphous carbon (such as a glassy carbon foam) can provide any advantageous properties, as it may conceivably have better properties than a simple carbon black catalyst.

Glassy carbon foam (GCF) is a special, hard, turbostratic carbon structure that is unique among the different carbon structures. It is known as a highly porous material with an outstanding specific surface area and high electrical and thermal conductivity [18,19]. Moreover, the structure can be modified by doping or with different coatings to improve mechanical, thermal, and other properties [20,21]. The number of oxygen-containing surface functional groups can also be increased, which can provide stronger interactions with other materials, such as catalytically active metals [22,23]. GCFs are mostly applied as thermal and acoustic insulation, adsorbent filters for molten metal alloys, a substrate for biological applications, and electrode material [24,25,26,27]. Glassy carbon foams may be a promising support for catalysts, owing to their large specific surface area and strong surface interactions due to the oxygen-containing surface functional groups. However, there is little literature about a catalytic application (except in electrocatalysis).

In this paper, a glassy carbon foam supported Pd catalyst was tested in nitrobenzene hydrogenation as a model reaction of nitro compound hydrogenation. The tests were carried out at different temperatures to examine temperature dependence and for kinetic calculations.

## 2. Materials and Methods

The glassy carbon foams were prepared as reported in our recent work [28]. In brief, the post-industrial waste polyurethane elastomer (Elastico Kft., Miskolc, Hungary) as a sacrificial template was immersed in 2.5 g/mL concentrated sucrose (Magyar Cukor Zrt., Koronás ^TM^, Kaposvár, Hungary) and 2.8% (*v*/*v*) dilute sulphuric acid solution (VWR International Kft., Debrecen, Hungary) to prepare the precursor. The waste foams were soaked in the sucrose solution for 12 h followed by atmospheric drying overnight and oven-drying at 383 K for 10 h. The impregnated polyurethane elastomers were directly activated in a CO_2_ atmosphere (Messer Group GmbH, Siegen, Germany) with a flow rate of 200 mL/min at 1273 K and a heating rate of 10 K/min with a dwell time of 100 min (denoted as ‘GCF-AC1’). In the other case, the impregnated foams were first carbonized at 1173 K at a heating rate of 10 K/min for 1 h in N_2_ (Messer Group GmbH, Siegen Germany), with a flow rate of 200 mL/min (denoted as GCF), followed by activation in CO_2_ in the above-mentioned activation condition (denoted as GCF-AC2).

The Pd nanoparticles were prepared using Pd(II)-nitrate dihydrate (Sigma-Aldrich, Budapest, Hungary; Pd(NO_3_)_2_ × 2 H_2_O, 40% Pd basis) as a precursor with water as a solvent, and hydrogen gas (Messer 4.5) for activation.

The glassy carbon foam samples (0.8988 g) were impregnated in a palladium-nitrate solution (0.1125 g/10 mL dist. water). The water evaporated from the solid phase after the samples were dried at 393 K overnight. The palladium-impregnated carbons were calcined in a nitrogen atmosphere (flowrate: 50 scm N_2_) at 673 K (30 min) after the samples were hydrogenated in hydrogen flow (50 scm H_2_) for one hour.

SEM images of the glassy carbon foams were fabricated by a Hitachi 4800 instrument and the samples were fixed with carbon tape rubber. Catalysts containing palladium were examined by high-resolution transmission electron microscopy (HRTEM, FEI Technai G^2^ electron microscope, 200 kV) to explore the morphology and particle size of the Pd nanoparticles. Sample preparation was performed by dropping an aqueous suspension of the catalyst samples on 300 mesh copper grids (lacey carbon, Ted Pella Inc., Redding, CA, USA). The diameters of the nanoparticles were measured on the HRTEM images, based on the original scale bar using ImageJ software.

X-ray diffraction (XRD) measurements were carried out with a Bruker D8 Advance diffractometer (Cu-Kα source, 40 kV and 40 mA, with Vantec 1 detector) to identify and quantify the palladium.

Identification of the surface functional groups was performed with a Bruker Vertex 70 Fourier transform infrared spectroscopy (FTIR) spectrometer. Measurements were applied in KBr pellets (5 mg sample in 250 mg KBr) in an interval of 400–4000 cm^−1^ and the resolution was 4 cm^−1^ next to a 16 min^−1^ scan rate.

Zeta potential measurements were carried out in the aqueous phase (2 mg sample in 50 mL distilled water) with Malvern Zeta Sizer Nano Zs equipment, using laser Doppler microelectrophoresis. Zeta potential calculation was performed based on the electrophoretic mobility measurement using Henry’s equation.

The hydrogenation of nitrobenzene (c = 0.25 mol/dm^3^ in methanolic solution) was carried out in a 200 mL Büchi Uster Picoclave reactor using 0.10 g catalyst. The pressure of hydrogenation was constant (20 bar), and the reaction temperature was set to 283, 293, 303, or 323 K. The rotational speed of agitation was 1000 rpm. Sampling occurred after the beginning of the reaction at 0, 5, 10, 15, 20, 30, 40, 60, 80, 120, 180, and 240 min. The quantitative analysis of the samples was carried out by an Agilent 7890A gas chromatograph coupled with an Agilent 5975C mass-selective detector. The separation was performed on an RTX-624 column (60 m × 0.25 mm × 1.4 μm). The injected sample volume was 1 μL at a 200:1 split ratio, while the inlet temperature was set to 473 K. Helium was the carrier gas with a constant flow (2.28 mL/min) and the oven temperature was set to 323 K for 3 min and then heated to 523 K, with a heating rate of 10 K/min, for another 3 min. The analytical standards of the main product, the by-products, and the intermediates’ standards originated from Sigma Aldrich (St. Louis, MO, USA) and Dr. Ehrenstorfer Ltd. (Hong Kong).

The efficiency of the catalyst was determined during the catalytic hydrogenation of nitrobenzene to aniline by calculating the conversion (X %) of nitrobenzene based on the following equation (Equation (1)):

(1) $$X\;\%=\frac{\mathrm{consumed}\;n_{\mathrm{nitrobenzene}}}{\mathrm{initial}\;n_{\mathrm{nitrobenzene}}}\;\cdot \;100\;$$

The process of nitrobenzene hydrogenation is interpretable as a first-order reaction [29,30,31]. Based on the initial and final nitrobenzene concentrations (c_0_ and c_k_, mol/dm^3^), the reaction rate constant (k) was calculated at different temperatures by non-linear regression based on the following equation (Equation (2)):

(2) $$c_{k}=c_{0}\cdot \;\exp\left(-k\cdot t\right)$$

Furthermore, the yield (Y %) of the aniline was calculated as follows (Equation (3)):

(3) $$Y\;\%=\frac{n_{\mathrm{aniline}}}{n_{\mathrm{nitrobenzene}\;}}\;\cdot \;100$$

where **n**_aniline_ and **n**_nitrobenzene_ are the corresponding chemical amounts of the compounds.

## 3. Results and Discussion

### 3.1. Characterization of Glassy Carbon Foam Catalyst Supports

**Figure 1:**
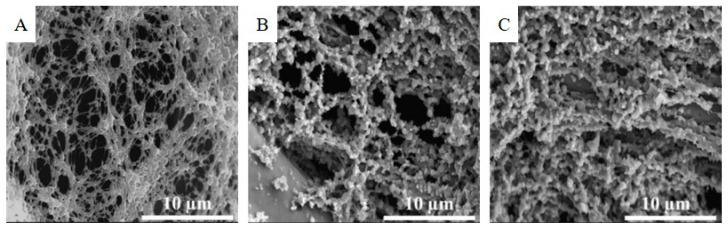
SEM pictures of the 3D structure of the glassy carbon foams, GCF (**A**), GCF-AC1 (**B**), and GCF-AC2 (**C**).

The 3D structure of the glassy carbon foam samples was examined by the SEM technique. The SEM picture of the GCF shows that a porous structure arose from the application of the polyurethane template during the synthesis (Figure 1A). The GCF sample prepared only by pyrolysis in a nitrogen flow at 1173 K resulted in a specific surface area of 370 m^2^/g. After heat treatment of the impregnated foams in a CO_2_ atmosphere at 1273 K, the structure of the carbon foam was changed (Figure 1B,C). The fibrous structure was missing in the case of the GCF-AC1; instead, spherical particles were visible in each case, which built up the three-dimensional carbon cluster. The structural change was noticeable due to the extreme increase in the specific surface area from 370 (GCF) to 2172 m^2^/g (GCF-AC1). The GCF-AC2 sample, which was treated in nitrogen at 1173 K and then activated in carbon-dioxide at 1273 K, did not show any structural changes compared with the GCF-AC1 (Figure 1C). However, the BET surface area of the GCF-AC2 was only 633 m^2^/g.

### 3.2. Characterization of the Palladium-Decorated Glassy Carbon Foam Samples

**Figure 2:**
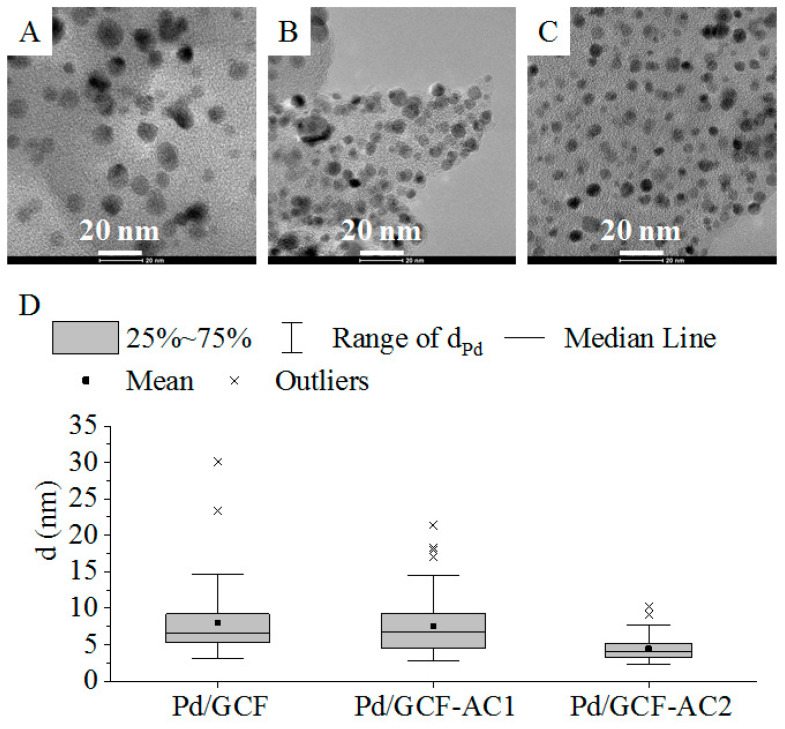
HRTEM pictures of sample Pd/GCF (**A**), Pd/GCF-AC1 (**B**) m and Pd/GCF-AC2 (**C**); statistics of particle size distribution (**D**).

The morphology and particle size of the palladium nanoparticles on the GCF supports were examined by the HRTEM technique. The HRTEM image of the three catalysts showed several approximately spherical palladium nanoparticles with small particle diameters (Figure 2A–C). The surface of the supports was covered homogeneously with Pd particles.

Size distribution analysis was applied to the three catalysts (Table 1). The particle size distribution in the case of the Pd/GCF and the Pd/GCF-AC1 catalysts was similar (Figure 2D), despite the varying BET surface area (370 and 2172 m^2^/g, respectively) of these catalyst supports. In contrast, the BET surface area of GCF-AC2 (633 m^2^/g) was less than that of GCF-AC1 (2172 m^2^/g), and yet the particle size of this catalyst was the smallest (Table 1).

An increased specific surface area enhances the force of adsorption interaction between the sorbent and the metal particles. However, other mechanisms play a role during sorption processes, namely ion exchange interactions, surface complexation or electronic interactions, and p-d hybridization. The strength of adsorption interactions influences the particle size by reducing surface migration, thereby increasing crystallite growth, which is why FTIR and zeta potential measurements are necessary.

**Figure 3:**
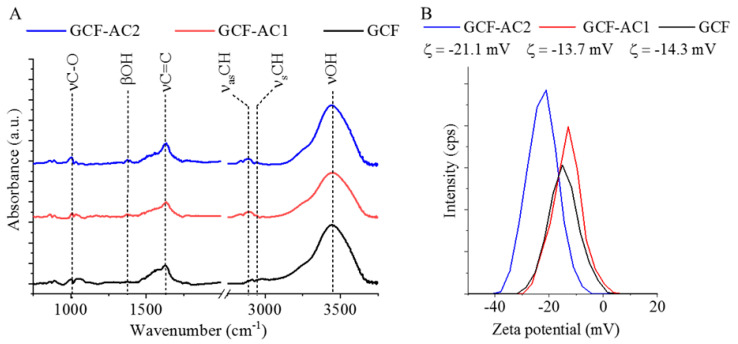
FTIR spectrum (**A**) and zeta potential distribution (**B**) of the Pd-decorated GCF samples.

FTIR is a useful technique for the identification of the surface functional groups (Figure 3A). On the spectrum of untreated GCF, a band at 1010 cm^−1^ was located that belonged to the C–O stretch vibration mode of the hydroxyl groups, while νOH vibration resulted in a band at 3440 cm^−1^. Peaks at 2885 and 2949 cm^−1^ wavenumbers signalled the presence of C–H stretching, which is typical for other GCF samples. The C=C stretching vibration resulted in adsorption peaks of 1630 cm^−1^ for all three samples. The βOH band was noticeable on the spectra of the GCF-AC1 and GCF-AC2 samples, which were activated by carbon-dioxide. The hydroxyl groups can be deprotonated, thus decreasing the zeta potential of the carbons. The most negative value was measured in GCF-AC2, which was −21.1 mV (Figure 3B). The oxidation improved the negative charge of the surface and the wettability of the GCF. The surface functional groups, mainly the –OH groups, advantageously influenced the anchoring of the platinum particles on the glassy carbon surface, which can decrease the particle size of palladium. Pd particle size was the smallest, 4.4 ± 1.6 nm, on the surface of GCF-AC2 (Table 2). Palladium ions prefer the –OH groups on carbon surfaces as adsorption sites and coordination or ion-exchange interaction thereby occurs between –OH groups and precious metal ions, which serve as nucleation sites for nanoparticles [30,31]. Moreover, the –OH groups can stabilize the anchoring of the metal cluster because, at these electron densities, hybridization between the precious metal d and carbon p orbitals are relatively more favored [32]. In this sense, the particle size of the Pd particles can be controlled by the concentration of –OH groups on the carbon support.

**Figure 4:**
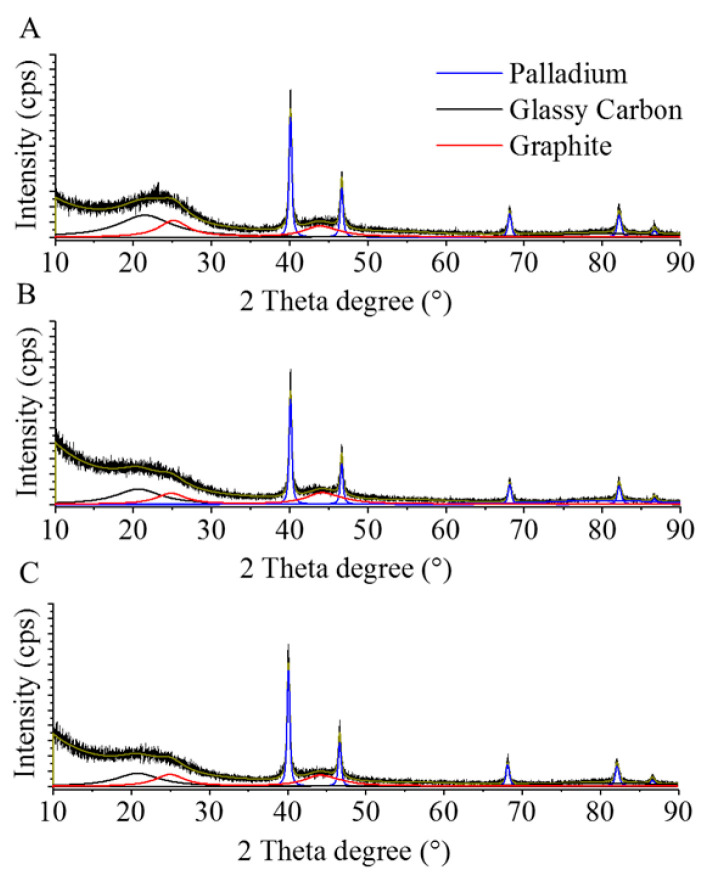
XRD pattern of the Pd/GCF (**A**), PD/GCF-AC1 (**B**), and Pd/GCF-AC2 (**C**) catalysts.

The presence of elemental palladium on the surfaces was confirmed by XRD measurements, as can be seen in Figure 4. On the diffractograms of the three catalysts, the identified reflections are attributed to the crystalline phases of Pd^0^ (Figure 4). On the XRD patterns, the Pd (111), Pd (200), Pd (220), Pd (311), and Pd (222) reflections are visible at 40.06°, 46.5°, 68.1°, 82.1°, and 86.5° two-theta degrees. Based on the XRD results, the hydrogenation of Pd ions during the catalyst preparation was efficient. Based on the pattern deconvolution by Rietveld refinement, we could not identify the oxidized form of the palladium. Two carbon phases were identified in the catalysts: glassy carbon and nanocrystalline graphite. The reflection at 20.7° two-theta degrees belonged to the glassy carbon. Two reflexions of the graphite were identified at 25.2° (002) and at 44.4° (101) two-theta degrees.

### 3.3. Catalytic Tests of the Glassy Carbon Foam Catalysts Containing Palladium

**Figure 5:**
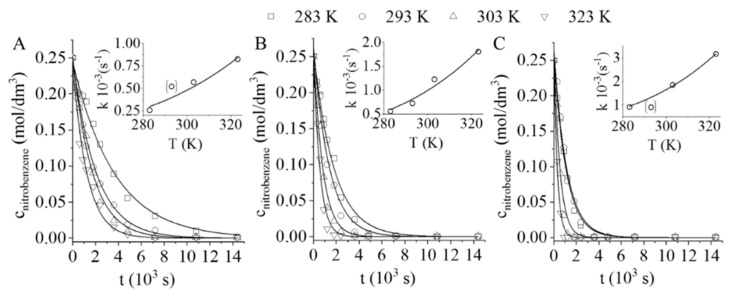
Nitrobenzene conversions vs. time of hydrogenation and the Arrhenius plots using Pd/GCF (**A**), Pd/GCF-AC1 (**B**), and Pd/GCF-AC2 (**C**) catalysts.

All catalysts were compared in terms of their catalytic activities in the hydrogenation of nitrobenzene to aniline. The total nitrobenzene conversion reached in all the cases is shown in Figure 5A–C. The non-treated, carbon-foam-supported palladium catalyst (Pd/GCF) resulted in a smaller hydrogenation rate than the other two catalysts; i.e., the 100% nitrobenzene conversion occurred only after 4 h at 50 bar hydrogen pressure and 283 K. By increasing the reaction temperature, the conversion was also increased and, at 323 K, the total nitrobenzene was converted in 2 h. The Pd/GCF-AC1 catalyst fabricated from the one-step activated (by only CO_2_) carbon foam showed higher activity than the non-treated GCF-supported catalyst (Figure 5B). The 100% nitrobenzene conversion was achieved within 2 h at 283 K (this required 4 h on the Pd/GCF catalyst). By increasing the temperature from 303 to 323 K, the reaction rate did not show any significant increase (Figure 5B). An intensified hydrogenation rate occurred on the Pd/GCF-AC2, activated in two steps (in N_2_ after CO_2_). In this case, the total quantity of the nitrobenzene was converted after only 60 min of hydrogenation at 283 K and higher temperatures (Figure 5C).

The non-linear regression method was used for the calculation of the reaction rate constants (k) [33]. In the case of the Pd/GCF-AC2, the samples had higher k values (Table 2).

By applying the reaction rate constants, the activation energy values were calculated using the Arrhenius plot. The rate constants were plotted as a function of temperature and the activation energy could then be calculated (Figure 5A–C inserts). The activation energy was 20.18 ± 4.9 kJ ∙ mol^−1^ in the case of the Pd/GCF. Similar results were observed for the other two catalysts, 21.43 ± 2.9 and 22.9 ± 0.9 kJ ∙ mol^−1^ (Table 3). Considering the standard deviation values, there was no significant difference between the activation energies. These values are lower than those found in the literature for catalysts containing Pd, Pt, or Ru, where activation energies vary between ~25 and ~45 kJ ∙ mol^−1^ [34,35,36].

**Figure 6:**
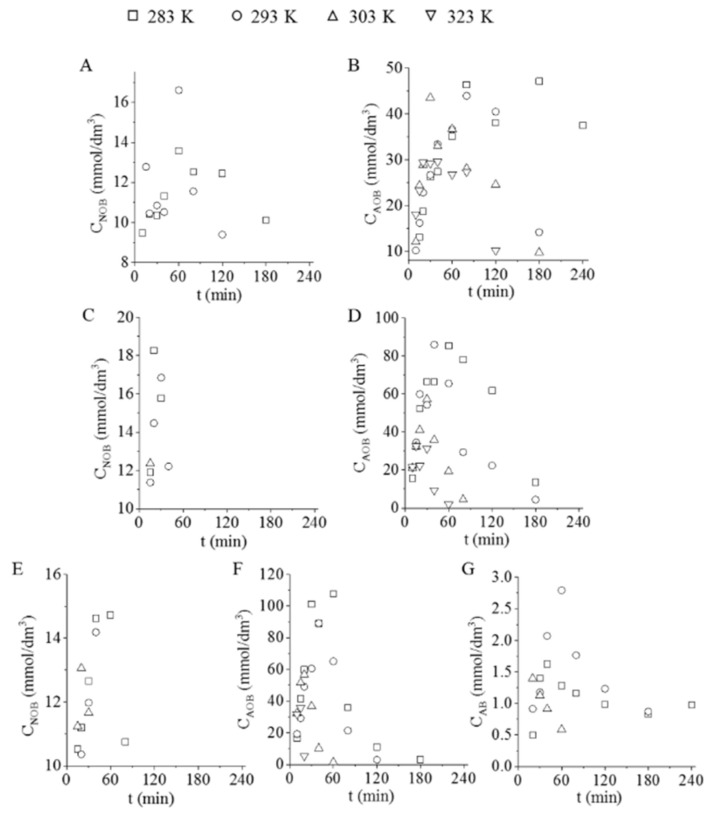
The concentration of the intermediates (nitrobenzene-NOB, azoxybenzene-AOB, and azobenzene-AB) in the reaction media vs. time of hydrogenation, using Pd/GCF (**A**,**B**), Pd/GCF-AC1 (**C**,**D**), and Pd/GCF-AC2 (**E**–**G**) catalysts.

The hydrogenation process of nitrobenzene towards aniline happens through the transformation of azoxybenzene (AOB), nitrosobenzene (NOB), and azobenzene (AB) intermediates (Figure 6A–G). In the case of the Pd/GCF catalyst, the total quantities of nitrosobenzene were transformed after 4 h, even at low temperatures (Figure 6A). However, the situation was different with azoxybenzene, as found in the samples fabricated at low-temperature (283 K) hydrogenation. In this case, the total amount of AOB was not converted to aniline (Figure 6B). In the case of the Pd/GCF-AC1 catalyst, the NOB conversion was much faster, as all NOB was reacted from the reaction media in less than 40 min (Figure 6C). After 4 h of hydrogenation, AOB was not detectable in the samples (Figure 6D). The observation was similar while using Pd/GCF-AC2 in the case of the NOB and AOB intermediates. Although a new intermediary azobenzene (AB) appeared, which was not transformed at low temperature (283 K), the temperature increase enhanced the conversion rate of AB.

Based on these results, it is clear that a temperature of 283 K was not high enough to achieve the total transformation of all intermediates.

**Figure 7:**
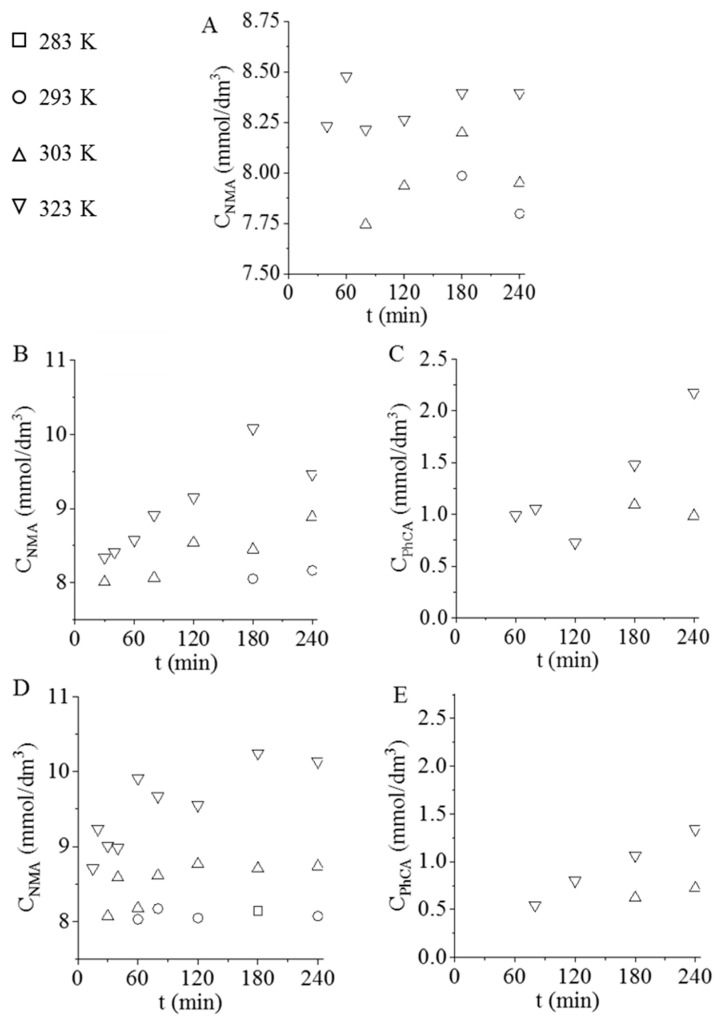
The concentration of by-products: N-methylaniline (NMA) and N-phenyl-cyclohexyl amine (PhCA) vs. time of hydrogenation at different temperatures using Pd/GCF (**A**), Pd/GCF-AC1 (**B**,**C**), and Pd/GCF-AC2 (**D**,**E**).

Several by-products can be formed during aniline synthesis but, in our experiments, the following by-products were identified in the samples: N-methylaniline (NMA) and N-phenyl-cyclohexylamine (PhCA). The formation of the above-mentioned by-products started after the intermediates were sold out. After exceeding the optimum synthesis time, methylation of the aniline and condensation and hydrogenation of the aromatic rings began and NMA and PhCA were formed. In the case of the Pd/GCF sample, NMA production was enhanced by increasing the temperature; it appeared at 293 K after 3 h, at 303 K after 2 h, and at 323 K after 40 min (Figure 7A). Through the application of Pd/GCF-AC1, which showed faster nitrobenzene conversion, NMA formed in a higher concentration than when using the Pd/GCF catalyst (Figure 7B). This tendency increased on Pd/GCF-AC2 catalyst, which resulted in a faster hydrogenation process than the other previous systems (Figure 7D). Pd/GCF-AC1 and Pd/GCF-AC2 at the higher temperatures (303 and 323 K) experienced the formation of PhCA in small concentrations, under 2.5 and 1.5 mmol/dm^3^, respectively (Figure 7C,E).

**Figure 8:**
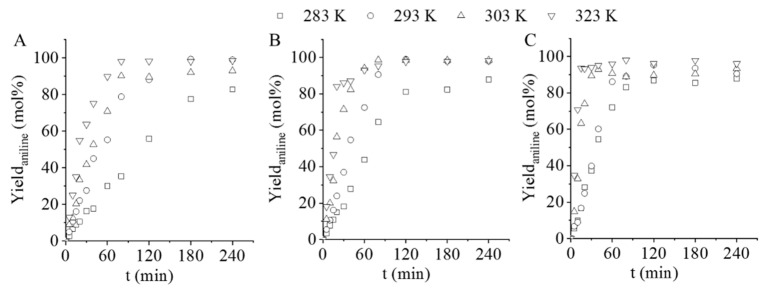
Aniline yield on the Pd/GCF (**A**), Pd/GCF-AC1 (**B**), and the Pd/GCF-AC2 (**C**) catalyst vs. time of hydrogenation.

The maximum aniline yield while using Pd/GCF catalyst was 99.2% and 98.4% at 293 and 323 K, respectively, after 180 min of hydrogenation (Figure 8A). At higher temperatures, especially at 303 and 323 K, NMA was formed in addition to aniline, which impaired the yield. At 283 K, the transformation of azoxybenzene was not completed, which also caused a decrease in yield. The Pd/GCF-AC1 resulted in 99.0%, 98.6%, and 97.7% aniline yield after 120 min of hydrogenation at 293, 303, and 323 K, respectively (Figure 8B). However, a slight decrease in the aniline yield was experienced while increasing the hydrogenation time. This may be due to the enhanced formation of the by-products (NMA, PhCA). The maximum aniline yield (98.0%) was reached after 80 min at 323 K when using Pd/GCF-AC2 (Figure 8C). A slight decline could be observed in this case as well, due to the formation of NMA and PhCA. Moreover, at low temperature (283 K), the azobenzene transformation to aniline was not completed.

These results suggest that, for each of the three catalysts, a maximum time should be set where the full conversion of nitrobenzene occurs without the formation of by-products. For our experimental setup, the optimal synthesis times and temperatures given at 20 bar pressure found for the three catalysts are summarized in Table 4.

**Figure 9:**
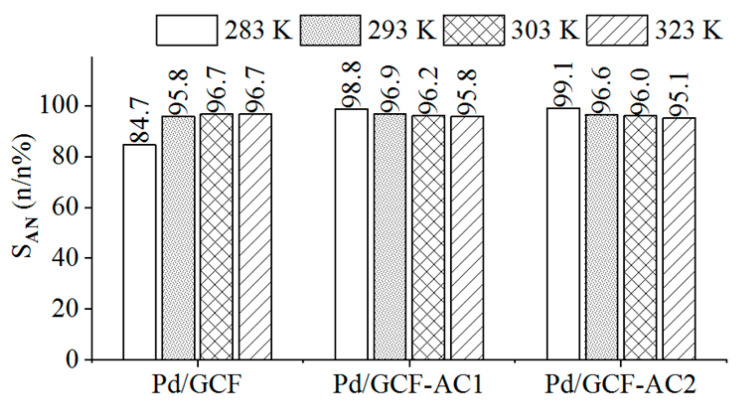
Selectivity of aniline on the Pd/GCF, Pd/GCF-AC1, and the Pd/GCF-AC2 catalyst at different temperatures.

The selectivity of aniline was above 98 n/n% in the case of the Pd/GCF-AC1 and Pd/GCF-AC2 catalysts at 323 K (Figure 9). The Pd/GCF-AC catalyst resulted 96.7 n/n% maximum selectivity at 303 and 323 K, which is also high. Based on the product selectivity, nitrobenzene conversion, and yield, it can be said that the glassy-carbon-supported palladium catalysts have high catalytic activity and are efficient catalysts. The excellent results could be explained by the strong interactions between the support and the Pd nanoparticles through surface functional groups. These groups also provided finer nanoparticles with better arrangements and, thus, increased the catalytic effectivity of the catalyst [37,38].

## 4. Conclusions

Highly efficient glassy carbon foam (GCF)-supported catalysts were developed with small palladium nanoparticles (d_Pd_ < 10 nm). The activation process of the glassy carbon samples with CO_2_ led to an increase in specific surface area from 370 to 2172 m^2^/g, which is a remarkable value. The CO_2_ activation of the carbons resulted in a decrease in zeta potentials, thus providing anchoring of the palladium ions on surfaces of the glassy carbon foam supports. Another advantage of the negative zeta potentials was that this enhanced the dispersibility of carbons in an aqueous solution of Pd-nitrate, which provided the homogenous coverage of GCF supports by Pd particles. Moreover, the negative zeta potential governed the small size of the Pd particles. The most negative surface potential was observed in the case of the Pd/GCF-AC2 catalyst, which was activated in two steps by using N_2_ after a CO_2_ atmosphere at 1273 K temperature. The Pd particle size was the smallest on the surface of the Pd/GCF-AC2 catalyst support. Another reason for the decreasing particle size was that the –OH groups stabilized the anchoring of the Pd cluster as, for the electron densities, hybridization between the palladium 4d and carbon 2p orbitals is relatively more favored. Moreover, the palladium ions prefer the –OH groups on carbon surfaces as adsorption sites, facilitating coordination or ion-exchange interaction between –OH groups and precious metal ions, which serve as nucleation sites for nanoparticles. All tested catalysts were efficient during the hydrogenation of nitrobenzene to aniline. The reaction rate constants (k) were increased in the following order: Pd/GCF < Pd/GCF-AC1 < Pd/GCF-AC2. A significant difference between the activation energies (Ea) was not experienced. Aniline yield was more than 98% and the complete nitrobenzene conversion was achieved in all cases. N-methylaniline (NMA) and N-phenyl-cyclohexylamine (PhCA) were formed as by-products in small concentrations (under 11 and 2.5 mmol/dm^3^, respectively). Formation of the by-products can be reduced or avoided by the careful choice of synthesis time and temperature; accordingly, the optimal synthesis parameters were suggested for all of the catalysts. Based on our results, it was found that the glassy carbon foam as catalyst support is effective for catalytic applications.

## Figures and Tables

**Table 1:** Statistics for the particle size of palladium.

	Pd/GCF	Pd/GCF-AC1	Pd/GCF-AC2
Mean with Std. (nm)	8.0 ± 4.3	7.6 ± 4.2	4.4 ± 1.6
Min. diameter (nm)	3.1	2.8	2.3
Max. diameter (nm)	30.1	21.4	10.2
1st quartile (nm)	5.3	4.5	3.3
3rd quartile (nm)	9.2	9.3	5.1

**Table 2:** Reaction rate constants (k) in s^−1^ dependence on the reaction temperature.

	283 K	293 K	303 K	323 K
Pd/GCF	2.5 × 10^−4^ ± 1.3 × 10^−5^	5.2 × 10^−4^ ± 1.6 × 10^−^^5^	5.7 × 10^−4^ ± 3.6 × 10^−5^	8.2 × 10^−4^ ± 6.3 × 10^−5^
Pd/GCF-AC1	5.6 × 10^−4^ ± 3.4 × 10^−5^	7.3 × 10^−4^ ± 5.9 × 10^−5^	1.2 × 10^−3^ ± 1.0 × 10^−4^	1.8 × 10^−3^ ± 1.3 × 10^−4^
Pd/GCF-AC2	9.7 × 10^−4^ ± 5.4 × 10^−5^	9.1 × 10^−4^ ± 7.2 × 10^−5^	1.9 × 10^−3^ ± 2.0 × 10^−4^	3.2 × 10^−3^ ± 1.6 × 10^−4^

**Table 3:** The activation energy (Ea) while applying the GCF-supported palladium catalysts.

	Pd/GCF	Pd/GCF-AC1	Pd/GCF-AC2
Ea (kJ ∙ mol^−1^)	20.18 ± 4.9	21.43 ± 2.9	22.9 ± 0.9

**Table 4:** Optimal synthesis parameters in case of each catalysts.

Cat. Type	Wt. (g)	c (mol/dm^3^)	p (bar)	T (K)	t (min)	Y_aniline_ (%)	Ea (kJ/mol)
Pd/GCF	0.10	0.25	20	293	180	99.2	20.18 ± 4.9
Pd/GCF-AC1				293	120	99.0	21.43 ± 2.9
Pd/GCF-AC2				323	80	98.0	22.90 ± 0.9

## Data Availability

Not applicable.

## References

[1] Rakitin M.Y., Doluda V.Y., Tereshchenkov A.Y., Demidenko G.N., Lakina N.V., Matveeva V.G., Sul’man M.G., Sul’man E.M. (2015). Investigating the catalytic hydrogenation of nitrobenzene in supercritical carbon dioxide using Pd-containing catalysts. Catal. Ind..

[2] Qu R., Macino M., Iqbal S., Gao X., He Q., Hutchings G.J., Sankar M. (2018). Supported bimetallic AuPd nanoparticles as a catalyst for the selective hydrogenation of nitroarenes. Nanomaterials.

[3] Wu S., Wen G., Zhong B., Zhang B., Gu X., Wang N., Su D. (2014). Reduction of nitrobenzene catalyzed by carbon materials. Cuihua Xuebao/Chin. J. Catal..

[4] Ma Y., Zhang L., Shi W., Niu Y., Zhang B., Su D. (2019). Facile-fabricated iron oxide nanorods as a catalyst for hydrogenation of nitrobenzene. Chin. Chem. Lett..

[5] Xiong W., Wang Z., He S., Hao F., Yang Y., Lv Y., Zhang W., Liu P., Luo H. (2020). Nitrogen-doped carbon nanotubes as a highly active metal-free catalyst for nitrobenzene hydrogenation. Appl. Catal. B Environ..

[6] Haber F. (1898). Gradual electrolytic reduction of nitrobenzene with limited cathode potential. Z. Elecktrochem..

[7] Gelder E.A., Jackson S.D., Lok C.M. (2005). The hydrogenation of nitrobenzene to aniline: A new mechanism. Chem. Commun..

[8] Peigney A., Laurent C., Flahaut E., Bacsa R.R., Rousset A. (2001). Specific surface area of carbon nanotubes and bundles of carbon nanotubes. Carbon N. Y..

[9] Cao Q., Xie K.-C., Lv Y.-K., Bao W.-R. (2006). Process effects on activated carbon with large specific surface area from corn cob. Bioresour. Technol..

[10] Popov V.N. (2004). Carbon nanotubes: Properties and application. Mater. Sci. Eng. R Rep..

[11] Ren X., Chen C., Nagatsu M., Wang X. (2011). Carbon nanotubes as adsorbents in environmental pollution management: A review. Chem. Eng. J..

[12] Oosthuizen R.S., Nyamori V.O. (2011). Carbon nanotubes as supports for palladium and bimetallic catalysts for use in hydrogenation reactions. Platin. Met. Rev..

[13] Fan G.Y., Huang W.J. (2015). Solvent-Free Hydrogenation of Nitrobenzene Catalyzed by Magnetically Recoverable Pt Deposited on Multiwalled Carbon Nanotubes. Synth. React. Inorg. Metal-Org. Nano-Met. Chem..

[14] Qu Y., Chen T., Wang G. (2019). Hydrogenation of nitrobenzene catalyzed by Pd promoted Ni supported on C 60 derivative. Appl. Surf. Sci..

[15] Jiang J., Zhai R., Bao X. (2003). Electrocatalytic properties of Cu-Zr amorphous alloy towards the electrochemical hydrogenation of nitrobenzene. J. Alloys Compd..

[16] Rodríguez-Ramos I., Reinoso F.R., Guerrero-Ruiz A., de Dios López-González J. (1986). Hydrogenation of CO and CO2 on carbon black-supported Ru catalysts. J. Chem. Technol. Biotechnol..

[17] Villano R., Acocella M.R., Guerra G. (2017). Oxidized Carbon Black as Catalyst for the Enamine Formation in Solvent-Free Conditions: A Green Strategy to Build the Benzodiazepine Scaffold. ChemistrySelect.

[18] Gancarczyk A., Macek W., Kołodziej A. (2019). Heat transfer phenomena of glassy carbon foams. Chem. Eng. Res. Des..

[19] Letellier M., Macutkevic J., Kuzhir P., Banys J., Fierro V., Celzard A. (2017). Electromagnetic properties of model vitreous carbon foams. Carbon N. Y..

[20] Xu L., Wu J.F., Bai S. (2012). Boron-doped glassy carbon fabricated by chemical vapor deposition. Xinxing Tan Cailiao/New Carbon Mater..

[21] Almajali M., Lafdi K., Prodhomme P.H., Ochoa O. (2010). Mechanical properties of copper-coated carbon foams. Carbon N. Y..

[22] Durkić T., Perić A., Laušević M., Dekanski A., Nešković O., Veljković M., Laušević Z. (1997). Boron and phosphorus doped glassy carbon: I. Surface properties. Carbon.

[23] Oishi S.S., Botelho E.C., Rezende M.C., Ferreira N.G. (2017). Structural and surface functionality changes in reticulated vitreous carbon produced from poly(furfuryl alcohol) with sodium hydroxide additions. Appl. Surf. Sci..

[24] Tadyszak K., Litowczenko J., Majchrzycki Ł., Jeżowski P., Załęski K., Scheibe B. (2020). Sucrose based cellular glassy carbon for biological applications. Mater. Chem. Phys..

[25] Friedrich J.M., Ponce-de-León C., Reade G.W., Walsh F.C. (2004). Reticulated vitreous carbon as an electrode material. J. Electroanal. Chem..

[26] Inagaki M., Qiu J., Guo Q. (2015). Carbon foam: Preparation and application. Carbon.

[27] Narasimman R., Vijayan S., Prabhakaran K. (2014). Carbon particle induced foaming of molten sucrose for the preparation of carbon foams. Mater. Sci. Eng. B Solid-State Mater. Adv. Technol..

[28] Udayakumar M., El Marabate B., Koós T., Szemmelveisz K., Kristály F., Leskó M., Filep Á., Géber R., Schabikowski M., Baumli P. (2021). Synthesis of activated carbon foams with high specific surface area using polyurethane elastomer template for effective removal of methylene blue. Arab. J. Chem..

[29] Yao H.-C., Emmett P.H. (1961). Kinetics of Liquid Phase Hydrogenation. II. Hydrogenation of Aromatic and Aliphatic Nitrocompounds Over A Colloidal Platinum Catalyst. J. Am. Chem. Soc..

[30] Plomp A.J., Su D.S., Jong K.P.D., Bitter J.H. (2009). On the nature of oxygen-containing surface groups on carbon nanofibers and their role for platinum depositionsan XPS and titration study. J. Phys. Chem. C.

[31] Zhong R.S., Qin Y.H., Niu D.F., Zhang X.S., Zhou X.G., Sun S.G., Yuan W.K. (2013). Effect of carbon nanofiber surface groups on oxygen reduction reaction of supported Pt electrocatalyst. Electrochim. Acta.

[32] Chutia A., Hamada I., Tokuyama M. (2014). A theoretical insight on the interaction between Pt nanoparticles and hydroxylated graphene nanoflakes. Surf. Sci..

[33] Wang C., Yang F., Yang W., Ren L., Zhang Y., Jia X., Zhang L., Li Y. (2015). PdO nanoparticles enhancing the catalytic activity of Pd/carbon nanotubes for 4-nitrophenol reduction. RSC Adv..

[34] Turáková M., Salmi T., Eränen K., Wärnå J., Murzin D.Y., Králik M. (2015). Liquid phase hydrogenation of nitrobenzene. Appl. Catal. A Gen..

[35] Easterday R., Sanchez-Felix O., Losovyj Y., Pink M., Stein B.D., Morgan D.G., Rakitin M., Doluda V.Y., Sulman M.G., Mahmoud W.E. (2015). Design of ruthenium/iron oxide nanoparticle mixtures for hydrogenation of nitrobenzene. Catal. Sci. Technol..

[36] Peureux J., Torres M., Mozzanega H., Giroir-Fendler A., Dalmon J.A. (1995). Nitrobenzene liquid-phase hydrogenation in a membrane reactor. Catal. Today.

[37] Kim J.H., Cheon J.Y., Shin T.J., Park J.Y., Joo S.H. (2016). Effect of surface oxygen functionalization of carbon support on the activity and durability of Pt/C catalysts for the oxygen reduction reaction. Carbon.

[38] Kim J.H., Yuk Y., Joo H.S., Cheon J.Y., Choi H.S., Joo S.H., Park J.Y. (2015). Nanoscale adhesion between Pt nanoparticles and carbon support and its influence on the durability of fuel cells. Curr. Appl. Phys..

